# Resveratrol attenuates endothelial oxidative injury by inducing autophagy via the activation of transcription factor EB

**DOI:** 10.1186/s12986-019-0371-6

**Published:** 2019-07-02

**Authors:** Xi Zhou, Jining Yang, Min Zhou, Yu Zhang, Yang Liu, Pengfei Hou, Xianglong Zeng, Long Yi, Mantian Mi

**Affiliations:** Research Center for Nutrition and Food Safety, Chongqing Key Laboratory of Nutrition and Food Safety, Institute of Military Preventive Medicine, Third Military Medical University (Army Medical University), NO.30 Gao Tan Yan Street, Shapingba District, Chongqing, 400038 People’s Republic of China

**Keywords:** Resveratrol, TFEB, Autophagy, Endothelial cells, Atherosclerosis

## Abstract

**Background:**

Endothelial oxidative injury is a key event in the pathogenesis of atherosclerosis (AS). Resveratrol (RSV) attenuates the oxidative injury in human umbilical vein endothelial cells (HUVECs). Autophagy is critical for the RSV-induced protective effects. However, the exact underlying mechanisms haven’t been completely elucidated. Thus, we aimed to explore the role of autophagy of the anti-oxidation of RSV and the underlying mechanism in palmitic acid (PA)-stimulated HUVECs.

**Methods:**

HUVECs were pretreated with 10 μM of RSV for 2 h and treated with 200 μM of PA for an additional 24 h. Cell viability, intracellular reactive oxygen species (ROS) and malondialdehyde (MDA) levels were estimated with a microplate reader and confocal microscope. Autophagosomes were analyzed by transmission electron microscopy, while lysosomes by confocal microscopy. The expression of transcription factor EB (TFEB) and related genes were quantified by qRT-PCR assay. Furthermore, TFEB levels, autophagy, and lysosomes were examined by western blot assay.

**Results:**

RSV pretreatment suppressed the PA-induced decline in cell viability and elevation in ROS and MDA levels in HUVECs. RSV pretreatment also increased LC3 production and P62 degradation while promoted the autophagosomes formation. However, 3-methyladenine (3-MA) treatment attenuated RSV-induced autophagy. RSV pretreatment upregulated the TFEB and TFEB-modulated downstream genes expression in a concentration-dependent manner. Additionally, in cells transfected with TFEB small interfering RNA, RSV-induced TFEB expression and subsequent autophagy were abolished. Meanwhile, the TFEB-modulated genes expression, the lysosomes formation and the RSV-induced anti-oxidation were suppressed.

**Conclusions:**

In HUVECs, RSV attenuates endothelial oxidative injury by inducing autophagy in a TFEB-dependent manner.

## Background

Atherosclerosis (AS) is the most serious threat to human cardiovascular health because it can lead to the coronary artery and cerebrovascular diseases, which is associated with a heavy economic burden [1, 2]. As a multifactorial disease, AS is induced by several cardiovascular risk factors, especially the dysfunction of vascular endothelial cells [3, 4]. Endothelial oxidative injury, induced by palmitic acid (PA), which promotes apoptosis and causes endothelial dysfunction, is a driving force in the initiation and development of AS [5, 6]. Thus, attenuating endothelial oxidative injury is the key to reversing the progression of AS.

Resveratrol (3, 4′, 5-trihydroxystilbene; RSV) is a naturally occurring polyphenolic compound that is mostly extracted from grapes and has been associated with multiple health benefits, such as anti-AS effects. According to the classic French paradox, one of the important reasons for the low incidence of cardiovascular diseases in France is that the French drink wine, in which RSV is the most effective antioxidant phytochemical [7]. We previously proved that RSV can attenuate endothelial dysfunction owing to its anti-oxidative bioactivity [8]. Nevertheless, the underlying mechanisms have not been fully clarified. Autophagy reportedly plays a key role in lipid catabolism and cellular clearance and affects various physiological processes [9–13]. Specifically, multiple research models have suggested that autophagy is critical for the RSV-induced protective effects [14, 15]. Thus, we wondered whether autophagy plays a key role in the RSV-induced anti-oxidative effects in human umbilical vein endothelial cells (HUVECs).

Although autophagy in response to metabolic cues has been studied for decades, the exact mechanisms mediating the autophagy induced by RSV and the related essential transcriptional regulation remain uncharacterized. Recent studies revealed that transcription factor EB (TFEB), which is a dominant regulator of the autophagy-lysosome pathway, also regulates organismal transcription and metabolism [16–18]. Moreover, increased TFEB activation may regulate autophagy induction and lysosomal biogenesis under starvation conditions, similar to the effects of caloric restriction. Additionally, TFEB activation may extend the lifespan of *C. elegans*, implying that increased longevity is regulated by nutrients [16]. A previous study showed that RSV can mimic the effects of caloric restriction and promote longevity [19]. Therefore, we hypothesized that the protective effect of RSV in response to endothelial oxidative injury occurs via TFEB-mediated autophagy. To prove this hypothesis, we studied the potential role of an RSV pretreatment in preventing PA-induced oxidative injury and in subsequently activating autophagy in HUVECs. Furthermore, we explored the expression of *TFEB* and downstream genes related to autophagy and lysosomal biogenesis, such as those encoding ATPase H+ transporting V0 subunit D1 (*ATP6V0D1*), lysosomal-associated membrane protein 1 (*LAMP-1*), cathepsin B (*CTSB*), microtubule-associated proteins 1A/1B light chain 3B (*MAP 1LC3B*), and UV resistance-associated gene (*UVRAG*). Additionally, based on a *TFEB* small interfering RNA (siRNA) transfection experiment, we investigated the role of TFEB in RSV-induced autophagy. This study demonstrated, for the first time, that RSV protects endothelial cells from oxidative injury by inducing autophagy in HUVECs, at least partially in a TFEB-dependent manner.

## Methods

### Chemicals and reagents

The culture medium of HyQ M199/EBSS (SH30351.01) and fetal bovine serum (SH30370.03) were purchased from HyClone Laboratories (Logan, UT, USA). Trans-resveratrol, dimethyl sulfoxide, PA, phosphate-buffered saline (PBS), 3-methyladenine(3-MA), the antibody of Histone H3 and LC3 were obtained from Sigma-Aldrich (St. Louis, MO, USA). A Cell Counting Kit (CCK-8; CK04) was purchased from Dojindo Laboratories (Dojindo, Kumamoto, Japan). Reactive Oxygen Species (ROS) Assay Kit, Lipid Peroxidation malondialdehyde (MDA) Assay Kit, Lyso-Tracker Red and Hanks’ Balanced Salt Solution (with Ca^2+^ & Mg^2+^) were obtained from the Beyotime Institute of Biotechnology. HRP-conjugated anti-mouse and anti-rabbit secondary antibodies were purchased from Invitrogen. Antibodies of P62, TEFB, and LAMP1 were obtained from Cell Signaling Technology, whereas, β-actin antibody, fluorescein isothiocyanate (FITC)-conjugated secondary antibody, TFEB siRNA and control siRNA were purchased from Santa Cruz Biotechnology, Inc. (Santa Cruz, CA, USA).

### Cell culture and treatment

According to the previous study, we isolated HUVECs from umbilical cord veins [8] and cultured with M199 medium added with 10% fetal bovine serum and 1% penicillin-streptomycin, at 37 °C and 5% CO_2_. Cells from 3 to 6 passages were adopted to the following experiments. During the logarithmic growth phase, cells were pretreated with10 μM of RSV for 2 h and then exposed to 200 μM of PA for an additional 24 h. Cells were also exposed with 3-MA (5 mM) for 1 h after the adding resveratrol for 2 h. This study was approved by the ethics committee of Army Medical University and consent was attained by all the involved patients.

### Cell proliferation

Cell proliferation was analyzed using a Cell Counting Kit-8 (Dojindo, Kumamoto, Japan) as previously described [20]. Briefly, PA was dissolved in 0.1 M NaOH at 70 °C to form a 100 mM PA solution firstly. Then, we dissolve it in 10% BSA solution and stirred it in a 55 °C water bath for about 3 h. 0.22 μm filter was used to sterilize the PA solution and save at − 20 °C to prepare a PA reserve liquid. Then, 8000 cells were seeded into 96-well microplates and then exposed to a series of PA concentrations (0, 100, 150, 200, 250 and 300 μM) for a series of time points (12, 16, 20 and 24 h). To detect the effect of RSV on PA-induced oxidative stress damage, cells were treated with various concentrations of RSV (0, 0.1, 1 and 10 μM) for 2 h, and subsequently exposed to 200 μM of PA for an additional 24 h. Next, 20 μL of CCK-8 solution was added to each well followed by 1–2 h incubation at 37 °C. A monochromator microplate reader was used to measure the absorbance (Molecular Devices, Sunnyvale, CA, USA) at 450 nm. Cell proliferation was calculated from the ratio of the optical density of the experimental cells to that of the control cells (set as 100%).

### Western blot analysis

The total cell lysate was analyzed by western blot analysis. Briefly, Equal amounts (50 μg) of proteins were resolved by 10% sodium dodecyl sulfate-polyacrylamide gel electrophoresis and the electroblotted onto polyvinylidene difluoride membranes for western blot analysis. Blots were probed with primary antibodies overnight at 4 °C. The primary antibodies are listed in Table 1. Specially, the nuclear protein and cytoplasmic protein were isolated respectively by Nuclear and Cytoplasmic Protein Extraction Kit (P0027, Beyotime, China) according to the instruction. The two fractions of proteins were tested by western blot to quantify the protein level of nuclear TFEB and cytosolic TFEB. The protein level ratio of nuclear TFEB to cytosolic TFEB was calculated by which the value of nuclear TFEB/Histone H3 divided by cytosolic TFEB/β-actin. Immune complexes were visualized using Immobilon Western Chemiluminescent HRP Substrate (Millipore, USA) and the signal was captured by Fusion FX (Vilber Lourmat, France). Densitometry analysis was computed using Image J software (NIH, MD, USA).

**Table 1:** Antibodies used for the western blot experiments

Antigen	Dilution	Supplier	Charge number
LC3	1:1000	Sigma	L7543
P62	1:1000	Cell Signaling Technology	5114
TFEB	1:1000	Cell Signaling Technology	37,785
LAMP1	1:1000	Cell Signaling Technology	9091
Histone H3	1:1000	Sigma	H0164
β-actin	1:1000	Cell Signaling Technology	4967

### RNA extraction and quantitative real-time polymerase chain reaction (qRT-PCR)

RNAiso Plus reagent (Takara Bio, Japan) was used to harvest total RNA according to the manufacturer’s instructions. qRT-PCR and data collection were performed by the qTower 2.2 real-time PCR system (Anakytik Jena, Germany) using SYBR Premix Ex Taq II (Tli RNaseH Plus) (Takara Bio, Japan). The primers for the targeted genes were produced by Sangon Biotech (Shanghai, China). Further, indicated genes were amplified according to the primers (Table 2). Relative fold-changes in gene expression were normalized to β-actin and analyzed by the 2^−ΔΔCt^ method.

**Table 2:** Sequences of primers used in quantitative RT-PCR

Target gene	Primer	Nucleotide sequence
*MAP1LC3B*	F	5′-AGCAGCATCCAACCAAAATC-3′
	R	5′-CTGTGTCCGTTCACCAACAG-3′
*ATP6V0D1*	F	5′-TTCCCGGAGCTTTACTTTAACG-3′
	R	5′-CAAGTCCTCTAGCGTCTCGC-3′
*UVRAG*	F	5′-GGCGTCTTCGACATCTTCGG-3′
	R	5′-GACGGTCTGGCATAATTCCAAA-3′
*CTSB*	F	5′-GAGCTGGTCAACTATGTCAACA-3′
	R	5′-GCTCATGTCCACGTTGTAGAAGT-3′
*LAMP-1*	F	5′-TCTCAGTGAACTACGACACCA-3′
	R	5′-AGTGTATGTCCTCTTCCAAAAGC-3′
*TFEB*	F	5′-ACCTGTCCGAGACCTATGGG-3′
	R	5′-CGTCCAGACGCATAATGTTGTC-3′
*β-actin*	F	5′-CGAGGCCCCCCTGAAC-3′
	R	5′-GCCAGAGGCGTACAGGGATA-3’

### siRNA assay

The siRNA for TFEB (human, sc-38,509), control siRNA (sc-44,230) and siRNA Transfection Reagent (sc-29,528) were obtained from Santa Cruz Biotechnology (USA). 100 nM of siRNA was applied to transfect HUVECs for 5–7 h, using Lipofectamine 2000 reagent (Invitrogen) following the manufacturer’s protocol. Then, the medium was exchanged with a fresh M199 medium. And the cells were incubated for another 24 h. As indicated, HUVECs were pretreated with 10 μM of RSV for 2 h and then exposed to 200 μM of PA for an additional 24 h. Subsequently, cells were collected for analysis. All the images were chosen from more than 3 samples and the evaluation process was double-blinded.

### ROS generation measurement

ROS levels were measured by Multi-Mode Microplate Readers (SpectramMax M5) and Confocal microscopy analysis. The determination of intracellular ROS production was measured with the fluorescent probes 2′,7′- dichlorofluorescein diacetate (DCFH-DA). HUVECs were incubated in a 96-well microplate at 8000 cells/well or 16,000 cells/dish. Then cells were dealt with different indicated treatments. Next, HUVECs were treated with 10 μM of DCFH-DA at 37 °C for 20 min, followed by washing with fresh fetal bovine serum-free media three times. The intracellular ROS levels were quantified by a Multi-Mode Microplate Readers or by confocal microscopic analysis (Radiance 2000, Bio-Rad, Hercules, CA). The intensity of emitted fluorescence following DCFH-DA treatment was associated with the quantities of ROS in the cell.

### Laser scanning confocal microscopy of HUVECs treated with lysotracker red

HUVECs were cultured at 16 × 10^3^ cells/dish overnight. Next, cells were treated to the illustrated treatments. Thereafter, cells were washed twice with complete media and incubated with 75 nM lysotracker red for 30 min. Subsequently, cells were washed with PBS three times and were visualized by the confocal microscope (Radiance 2000, Bio-Rad, Hercules, CA). Excitation of lysotracker red was at 577 nm and fluorescence emission was at 590 nm.

### MDA assay

For lipid peroxidation assay, the Lipid Peroxidation MDA assay kit (Beyotime, China) was used to access MDA generation. Based on the manufacturer’s procedure, total cell lysate was prepared, and the protein concentration was measured using a BCA assay (Beyotime, China). The MDA levels were quantified by Multi-Mode Microplate Readers (SpectramMax M5) at 532 nm.

### Transmission electron microscopy

HUVECs were harvested and fixed for 2 h, post-fixed with 1% OsO_4_ for 1.5 h, washed, and stained in aqueous uranyl acetate for 1 h. The samples were then washed again, dehydrated with graded alcohol, and embedded in Epon-Araldite resin (Canemco & Marivac, Lakefield, Quebec, Canada). Ultrathin sections were obtained by an ultramicrotome (Reichert-Jung, Inc., Cambridge, UK), counterstained with 0.3% lead citrate, and visualized on a transmission electron microscope (EM420, Koninklijke Philips Electronics N.V., Amsterdam, The Netherlands).

### Statistical analyses

The statistical analysis was conducted with the t-test and one-way analysis of variance by SPSS 13.0 statistical software (SPSS Inc., IBM, USA). A two-sided *p*-value < 0.05 was regarded as the statistical significance and the Tukey-Kramer post-hoc test was applied if *p* < 0.05. Quantitative data are presented as means ± standard deviation (X ± SD). All the experiments were repeated independently at least three times.

## Results

### RSV attenuated PA-induced oxidative injury in HUVECs

**Fig. 1:**
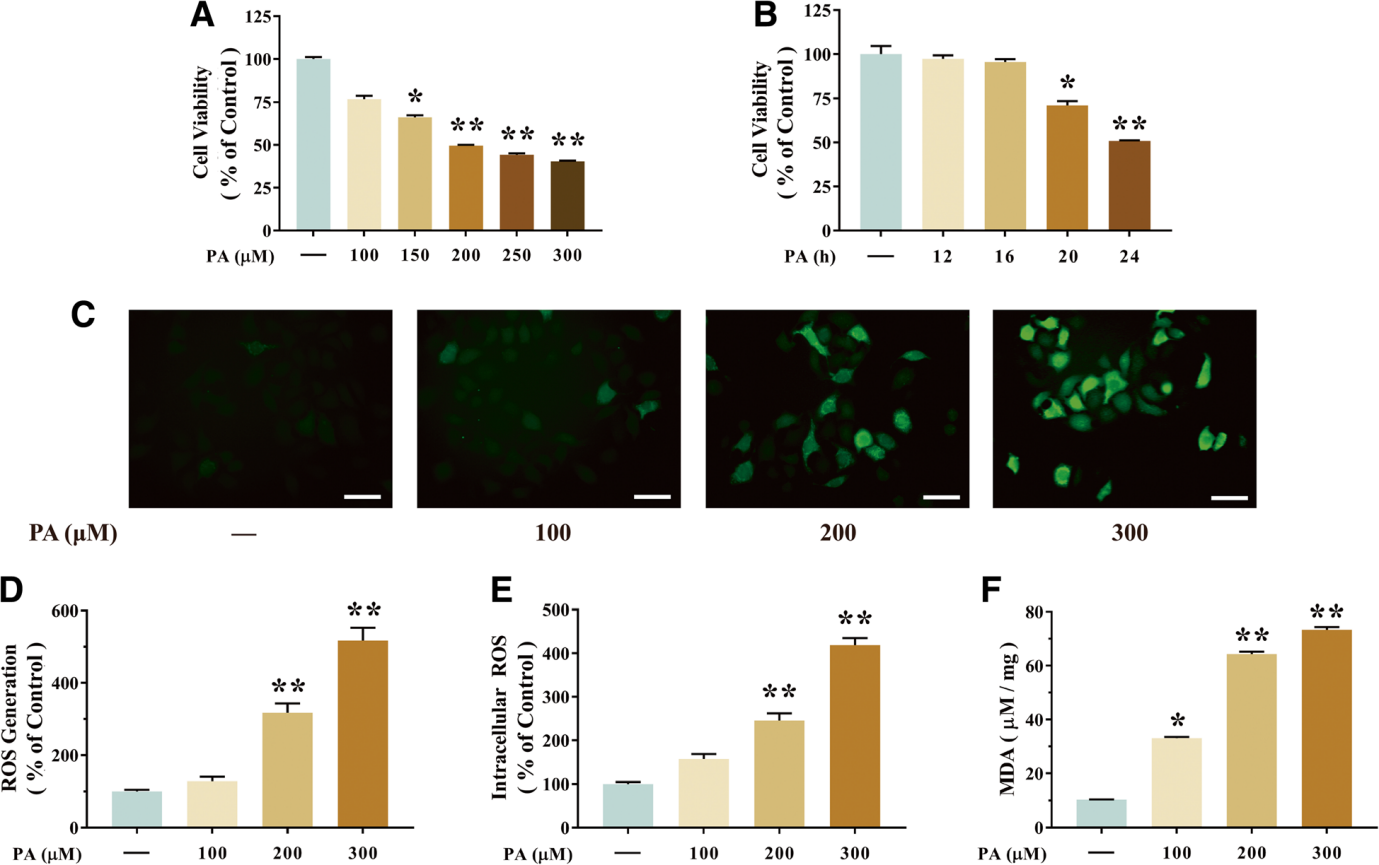
PA induces oxidative stress in HUVECs. **a** Cells were incubated with different concentrations (100, 150, 200, 250 and 300 μM) of PA. Cell viability was measured by CCK-8 detection kit as described in the Materials and methods section. **b** Cells were incubated with different time (12, 16, 20 and 24 h) of PA. Cell viability was measured by CCK-8 assay. **c**-**e** Intracellular ROS levels were estimated using a probe DCFH-DA by confocal microscopy (**c**) and The bar charts show quantification of the ROS generation levels expressed as the fold change (**d**), and by microplate reader, fluorescence was read at 485 nm for excitation and 520 nm for emission (**e**). **f** The levels of MDA were detected using an MDA detection kit. Values are presented as means ± SD (*n* = 6); *p < 0.05, **p < 0.01 compared with control groups

**Fig. 2:**
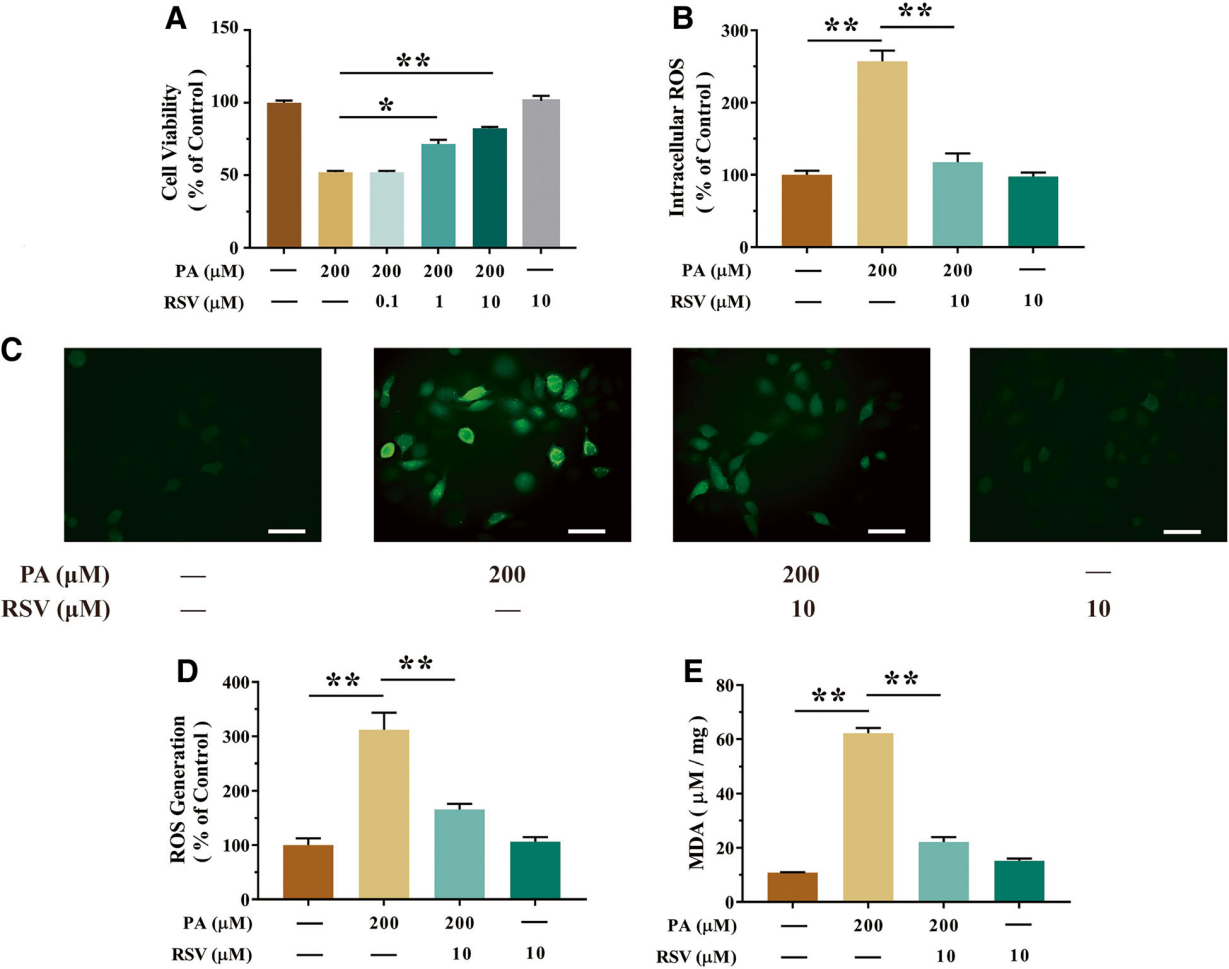
RSV inhibited PA-induced injury in HUVECs. **a** Cells were pretreated with different concentrations (0.1, 1 and 10 μM) of RSV for 2 h. Then, 200 μM of PA was added for an additional 24 h period. Subsequently, cell viability was measured using a CCK-8 detection kit, as described in the Materials and methods section. **b**-**d** Cells were pretreated with 10 μM of RSV for 2 h. Then, 200 μM of PA was added for an additional 24 h period. The intracellular ROS levels were estimated using the probe DCFH-DA by a microplate reader (**b**). Also, the intracellular ROS levels were estimated by confocal microscopy (**c**) and the bar charts show quantification of the ROS generation levels expressed as the fold change (**d**). **e** The levels of MDA were detected using an MDA detection kit. Values are presented as means ± SD (*n* = 5); *p < 0.05, **p < 0.01 compared between the marked groups

In accordance with previous observations, HUVECs viability was decreased by PA in a concentration-dependent and time-dependent manner in HUVECs. Cell viability showed 50.38% decline when treated with at least 200 μM of PA than that of 0 μM of PA (*p*<0.01, Fig. 1a). After treatment with 200 μM of PA for 24 h, the cell viability of HUVECs was significantly decreased which was 49.08% less than that of the initial time point (*p*<0.01, Fig. 1b). As shown in Fig. 2a, the protective function of RSV at various concentrations (0.1, 1 and 10 μM) was validated by inhibiting PA-induced decreasing cell proliferation in HUVECs. Specially, the precondition of 10 μM of RSV for 2 h could elevate the cell viability significantly which is 58.29% more than that of the PA-treated HUVECs (*p*<0.01, Fig. 2a). Consistently, intracellular ROS production was the 157.17% higher than when treated with 200 μM of PA than blank group by microplate reader (*p*<0.01, Fig. 2b), which was 212.16% higher than that of blank group by confocal microscopy (*p*<0.01, Fig. 2d), The MDA level was also 473.44% elevated by 200 μM of PA than that of blank group (*p*<0.01, Fig. 2e). In contrast, ROS was suppressed by precondition with 10 μM of RSV in PA-treated HUVECs, which was 54.29% less by a microplate reader (*p*<0.01, Fig. 2b) and 46.88% less by confocal microscopy than that of PA alone (*p*<0.01, Fig. 2d). MDA was also significantly decreased by precondition with 10 μM of RSV for 2 h, which is 74.35% less than that of the PA-treated HUVECs (*p*<0.01, Fig. 2e). All these data indicated that RSV pretreatment (especially at a concentration of 10 μM) for 2 h attenuated PA-induced injury in HUVECs.

### RSV attenuated PA-induced injury through stimulating autophagy in HUVECs

**Fig. 3:**
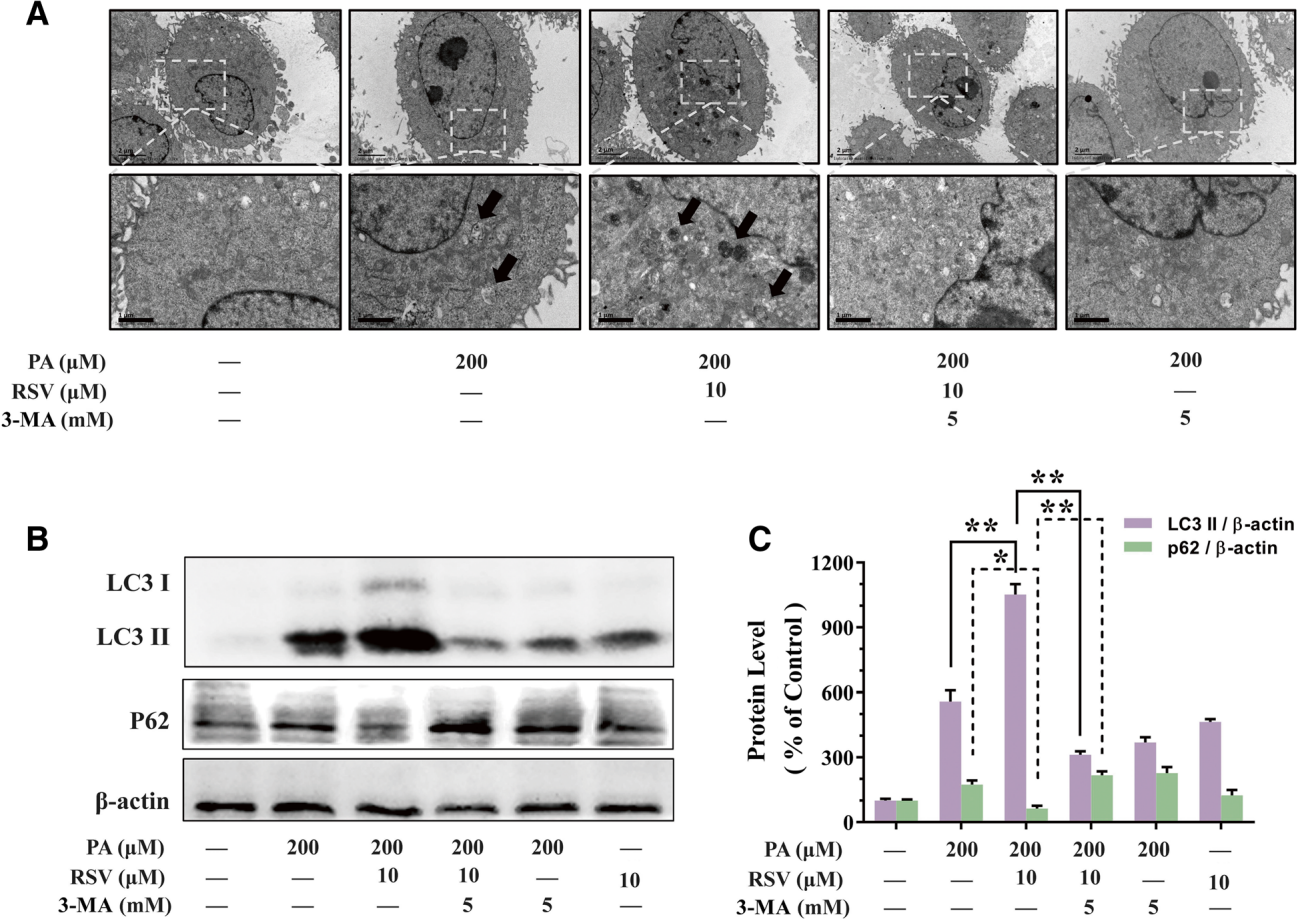
RSV attenuated PA-induced injury by inducing autophagy in HUVECs. HUVECs were pretreated with RSV (10 μM for 2 h) in the presence or absence of 3-MA (5 mM for 2 h), which we used to inhibit RSV-induced autophagy, followed by treatment with PA (200 μM for another 24 h). **a** Autophagosome formation was tested by TEM analysis after treatment as above. Arrows indicate the autophagosomes. Bar charts show the number of autophagosomes per cells of each experimental group. **b** The protein expression of LC3 II and P62 were analyzed by western blotting. **c** Bar charts show the quantification of endogenous LC3 II and P62. Values are presented as means ± SD (*n* = 3); *p < 0.05, **p < 0.01 compared between the marked groups

As shown in Fig. 3a, transmission electron microscopy analysis showed RSV pretreatment increased autophagosomes formation (*p*<0.05). Then, the expression of LC3 II (a precise marker of autophagy) and p62 (a key adaptor protein involved in selective autophagy) expression were analyzed by western blot. As shown in Fig. 3b and c, when treated with PA alone, the expression of LC3 II and P62 were both increased (*p*<0.05), indicating that autophagic flux was suppressed by PA. However, RSV pretreatment markedly resulted in 88.64% upregulation of LC3 II expression and 64.43% downregulation of P62 level in PA-treated HUVECs than that of the group of PA alone (*p*<0.05, Fig. 3b and c), which indicated that RSV activating autophagy in PA-stimulated HUVECs. Furthermore, pretreatment of 3-MA, an inhibitor of autophagy in the early stage, suppressed RSV-induced autophagosome formation (Fig. 3a), as well as, suppressed the increased expression of LC3-II and decreasing expression of P62 (*p*<0.01, Fig. 3b and c). Consequently, these data indicated that RSV attenuated endothelial oxidative injury in an autophagy-dependent manner.

### RSV increases TFEB activity in HUVECs

**Fig. 4:**
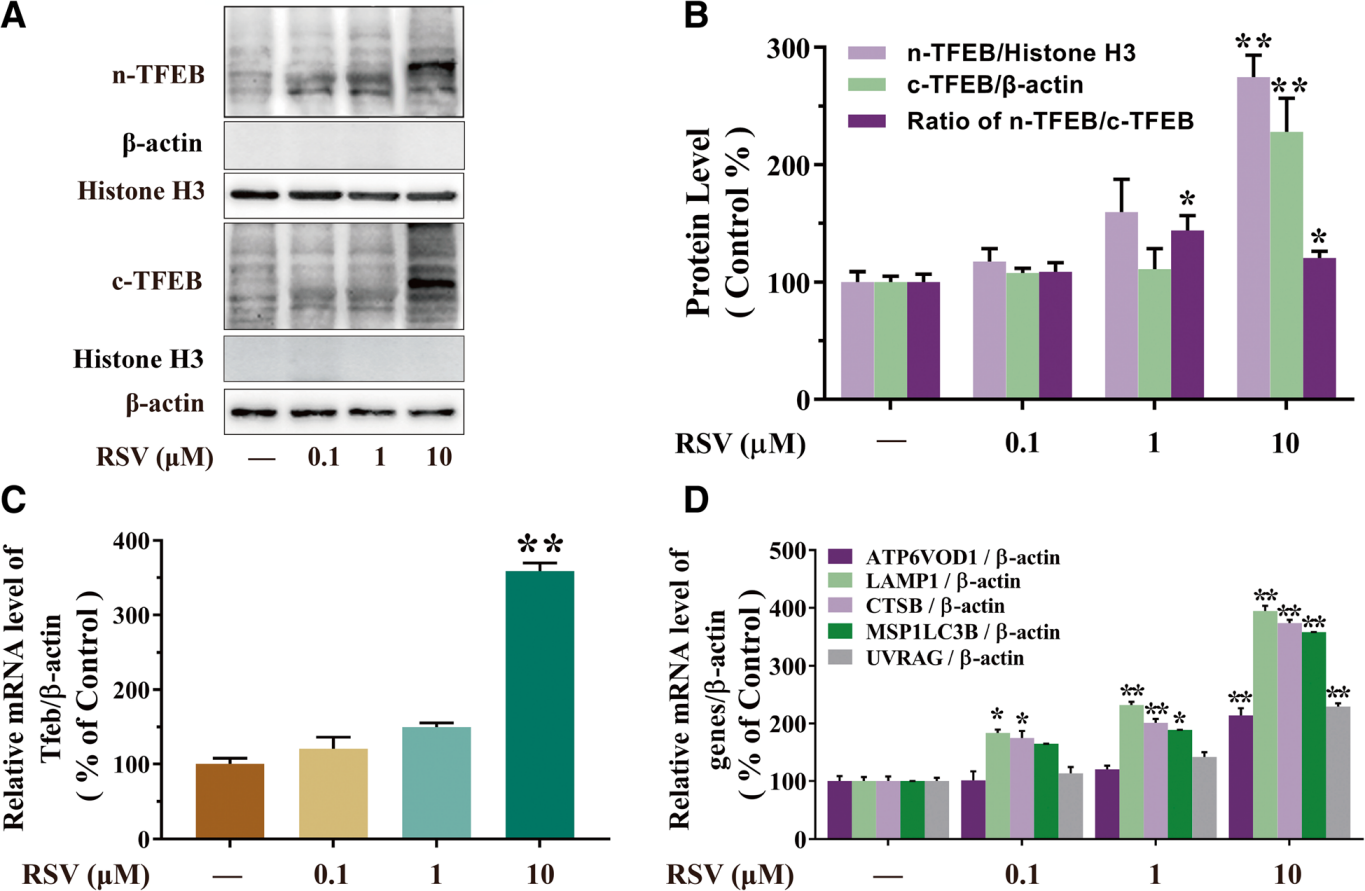
RSV increases TFEB activity in HUVECs. HUVECs were treated with different concentrations (0.1, 1, 10 μM) of RSV for 2 h. **a** The levels of nuclear and cytoplasmic TFEB were analyzed by western blotting, Histone H3 and β-actin were the standards for protein loading, respectively. **b** The bar graph shows the protein level quantification of nuclear TFEB (n-TFEB), cytoplasmic TFEB (c-TFEB) and the ratio of the endogenous nuclear TFEB to cytoplasmic TFEB. **c** The mRNA level of TFEB was then analyzed by qRT-PCR. **d** The mRNA levels of TFEB target genes were measured using qRT-PCR. Values are presented as means ± SD (*n* = 3); *p < 0.05, **p < 0.01 compared with control groups

TFEB is an important transcription factor of the MiT/TFE family that regulates lysosomal biogenesis and autophagy [21]. Many downstream genes of TFEB, including ATP6V0D1, LAMP1, CTSB, MAP 1LC3B and UVRAG, are involved in lysosome-associated processes and substrate degradation [22]. The activation of TFEB could be examined based on that its active form is located in cell nuclei while its inactive form is in the cytoplasm. As shown in Fig. 4 a and b, the protein expression of nucleus-TFEB and cytoplasm-TFEB induced by RSV with different concentrations (0.1, 1 and 10 μM) were analyzed by western blotting. We found that RSV exposure could activate TFEB, especially elevated the nucleus-TFEB expression. 10 μM of RSV markedly resulted in the 174.54% upregulation the protein expression of nucleus-TFEB and 128.19% upregulation of cytoplasm-TFEB than that of the blank group (*p*<0.01, Fig. 4b) in HUVECs. Meanwhile, the ratio of the nucleus- to cytoplasm-TFEB was 20.3% elevated by 10 μM of RSV than that of the blank group (*p*<0.05, Fig. 4b) in HUVECs. Furthermore, we examined the mRNA expression of TFEB, which presented comparable results in Fig. 4c. The mRNA expression of TFEB was 258.96% upregulated by the treatment of RSV than that of the blank group (*p*<0.01, Fig. 4c). Subsequently, our study showed 10 μM of RSV increased the nucleus translocation of TFEB along with an increased expression of TFEB-responsive genes. As we predicted, RSV increased the mRNA expression of ATP6V0D1, LAMP1, CTSB, MAP 1LC3B and UVRAG significantly (Fig. 4d). In general, these data shown that RSV motivated TFEB to translocate into the nucleus, elevated TFEB expression and activating TFEB-downstream genes related to autophagy and lysosomal biosynthesis.

### RSV-induced autophagy in PA-stimulated endothelial cells in a TFEB-dependent manner

**Fig. 5:**
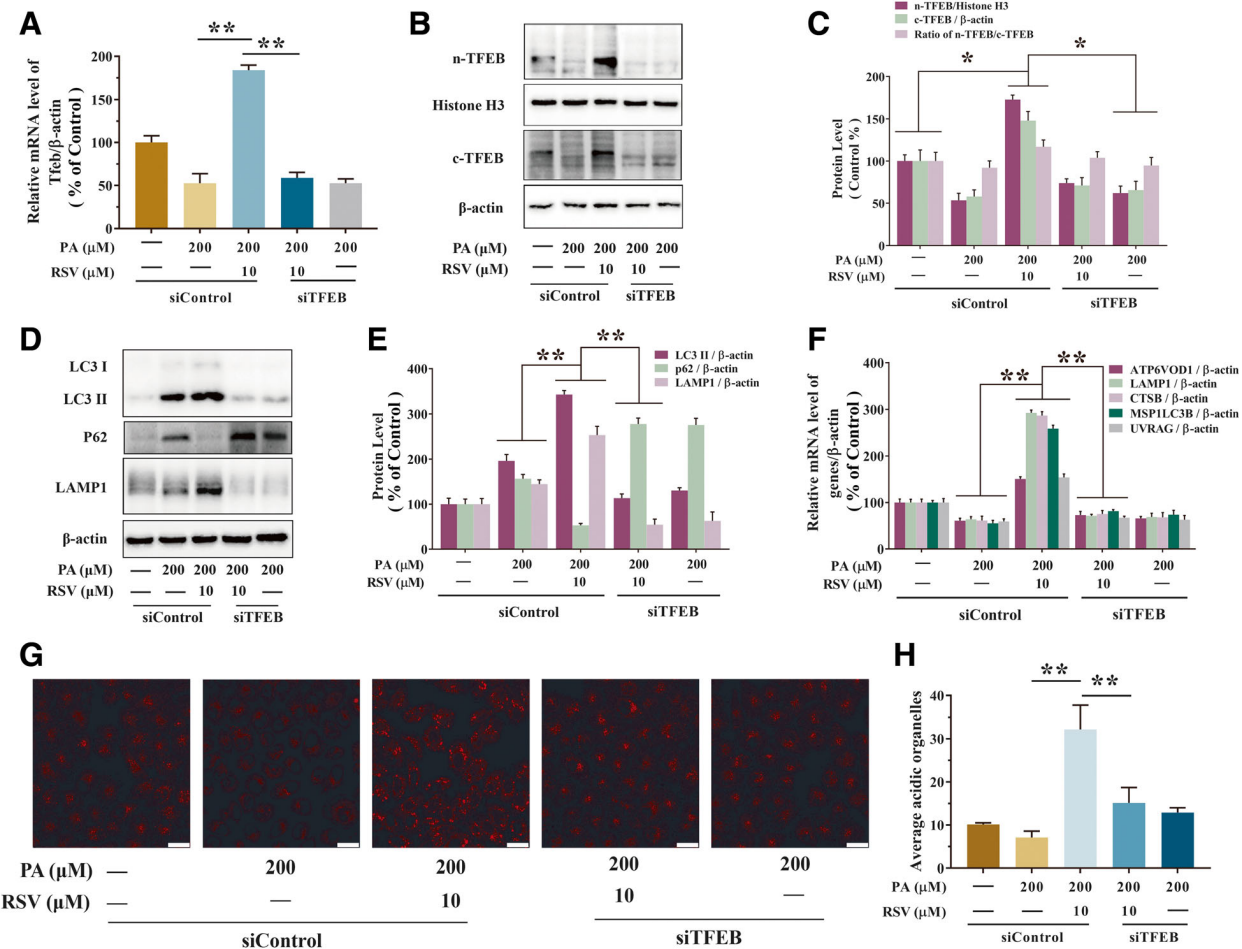
RSV stimulated autophagy through activating TFEB in PA-treated HUVECs. Cells were pretreated with RSV (10 μM) for 2 h, then treatment with PA (200 μM) for another 24 h. Furthermore, TFEB was knocked-down by siRNA transfection in HUVECs as described in the Materials and methods section. **a** The mRNA levels of TFEB was detected in HUVECs by using qRT-PCR. **b**-**c** The bar graph shows the protein level quantification of nuclear TFEB (n-TFEB), cytoplasmic TFEB (c-TFEB) and the ratio of the endogenous nuclear TFEB to cytoplasmic TFEB were analyzed by western blotting (**b**) and the ratio displayed by bar graph (**c**). **d**-**e** The protein levels of LC3 I, LC3 II, P62 and LAMP1 were analyzed by western blotting (**d**) and quantification displayed by bar graph (**e**). **f** The mRNA levels of TFEB target genes were measured using qRT-PCR. **g** The cells were incubated with lysotracker red (50 nM, 15 min, at 37 °C) and immediately visualized by confocal microscopy. **h** The average LTR fluorescence was expressed as the MFI using IPP 6.0 software. Values are presented as means ± SD (*n* = 3); *p < 0.05, **p < 0.01 compared between the marked groups

**Fig. 6:**
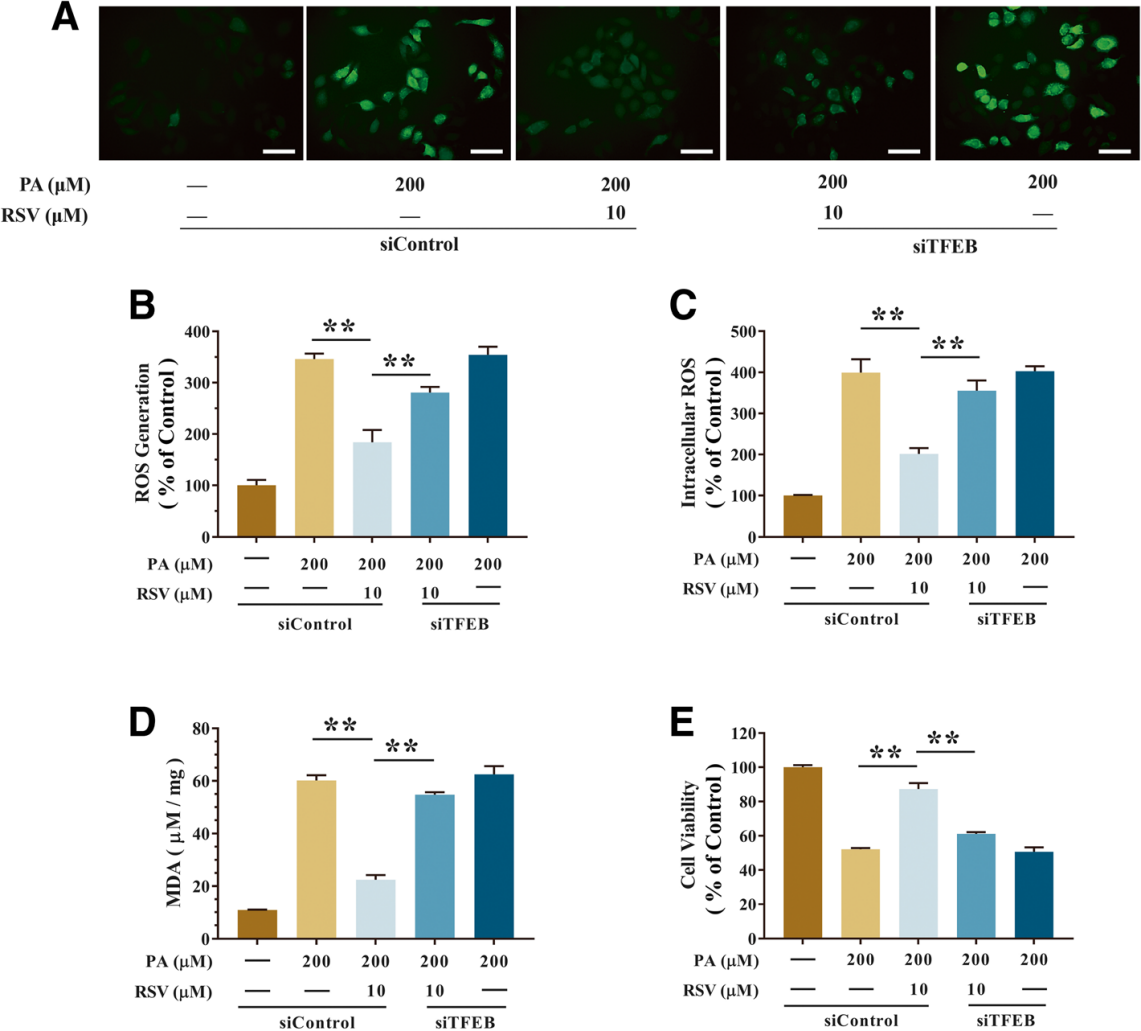
RSV attenuated oxidative injury through stimulating autophagy in a TFEB-dependent manner in PA-treated HUVECs. Cells were pretreated with RSV (10 μM) for 2 h, then treatment with PA (200 μM) for another 24 h. Furthermore, TFEB was knocked-down by siRNA transfection in HUVECs as described in the Materials and methods section. **a**-**c** Intracellular ROS levels were estimated using a probe DCFH-DA by confocal microscopy (**a**) and bar charts show quantification of the ROS generation levels expressed as the fold change (**b**), and by microplate reader, fluorescence was read at 485 nm for excitation and 520 nm for emission (**c**). **d** The levels of MDA were detected using an MDA detection kit. **e** The cell viability was measured using a CCK-8 detection kit, Values are presented as means ± SD (*n* = 6); *p < 0.05, **p < 0.01 compared between the marked groups

To further examine the capability of TFEB in RSV-induced autophagy, TFEB-specific siRNA (*siTFEB*) was transfected as described in the Materials and methods section, TFEB mRNA expression was significantly upregulated by RSV in PA-stimulated HUVECs, while it was knocked-down by TFEB siRNA transfection (Fig. 5a). The mRNA expression of TFEB was 248.55% upregulated by the RSV precondition in the PA-treated HUVECs than that of the group of PA alone (*p*<0.01, Fig. 5a). However, after treatment of TFEB siRNA, it resulted in 57.32% downregulation of n-TFEB (*p*<0.01, Fig. 5c) and in 11.05% downregulation of the ratio of n-TFEB to c-TFEB (*p*<0.05, Fig. 5c) in the PA-treated HUVECs than that of the group of RSV precondition. Meanwhile, the expression of RSV-induced upregulate of nuclear-TFEB, cytoplasmic-TFEB, LAMP1 and downregulate of P62 were significantly inhibited by *siTFEB* transfection in PA-treated HUEVCs (Fig. 5b-e). Furthermore, RSV-induced gene expression of lysosomal biosynthesis and autophagy was decreased by suppression of TFEB (Fig. 5f). In addition, confocal microscopy analysis exhibited that RSV exposure increased lysosomes in PA-treated HUVECs. And the function of RSV was 76.25% downregulated by *siTFEB* in PA-treated HUVECs than that of the group of RSV precondition (*p*<0.01, Fig. 5g and h). Further, the protective function of RSV in high levels of ROS and MDA caused by PA was also inhibited by *siTFEB* (Fig. 6a-d). As well, the ROS generation was significantly elevated by *siTFEB* in PA-treated HUVECs, which is 52.83% more by confocal microscopy (*p*<0.01, Fig. 6a and b) and 76.51% more by a microplate reader than the group of RSV precondition (*p*<0.01, Fig. 6c). Meanwhile, the CCK-8 analysis found that RSV resulted in the 29.93% upregulation of cell viability when inhibited by *siTFEB* in PA-treated HUEVCs than that of the group of RSV precondition (*p*<0.01, Fig. 6e). These results demonstrated that RSV reduced PA-induced oxidative stress by activating the TFEB-dependent autophagy pathway.

## Discussion

In this study, we provide evidence, for the first time, that RSV attenuates endothelial oxidative injury in HUVECs by inducing autophagy, partly by activating the TFEB signaling pathway. Specifically, we observed that (1) RSV can suppress oxidative stress and increase the viability of endothelial cells via autophagy induction and (2) the protective autophagy induced by RSV in HUVECs depends on a TFEB pathway.

Oxidative stress is regarded as the main inducer of endothelial dysfunction, which is the initial and crucial pathological process of AS [23]. In fact, all risk factors for cardiovascular diseases, such as high blood pressure, diabetes, and smoking, as well as the cardiovascular disease itself, are associated with increased ROS production in the vessel wall, which ultimately leads to oxidative stress [24, 25]. In this study, an endothelial oxidative stress model was established with PA, which is the main saturated free fatty acid in the bloodstream. Previously, PA is widely used to establish injury model in many metabolic diseases, including insulin resistance [26] and oxidative stress [27]. Particularly, PA has been used to treat ECs to induce endothelial oxidative stress as previously reported, which were consistent with our results. For example, PA reportedly induces oxidative injury and causes endothelial dysfunction. Jiang H et al. found that PA promotes the apoptosis of endothelial progenitor cells via p38 and JNK mitogen-activated protein kinase pathways [6]. Staiger et al. found that PA can induce vascular inflammation by increasing IL-6 production in human coronary artery endothelial cells [28]. In addition, Cacicedo et al. found that PA can induce apoptosis in cultured bovine retinal pericytes by increasing oxidative stress [29]. In contrast, the model of insulin resistance induced by PA was generally reported in the studies of the skeletal muscle system. Thus, we consider endothelial cells exposed to PA represent a suitable model for exploring the anti-oxidative mechanism of RSV.

Several pharmacological treatments for AS have been proposed, but few have exhibited both efficacy and long-term safety. Several pieces of evidence indicate that phytochemicals, which are abundant and available in the daily diet, can protect the cardiovascular system against AS by alleviating endothelial oxidative injury [30]. Specifically, the ameliorative effects of RSV on AS-associated oxidative stress have been confirmed in several studies [31–33]. Furthermore, RSV can protect endothelial cells against AS by decreasing ROS production, increasing NO production, and increasing the production of inflammatory factors [34, 35]. Therefore, we believe that the protective effects against AS are mainly related to the antioxidant activities of RSV. Due to the low bioavailability of RSV in vivo, the serum concentration of unmetabolized RSV generally peak in the sub- to the low-micromolar range and the peak concentration reached in the first 30 min~ 2 h. In our previous study, we found resveratrol was tolerated by endothelial cells which exhibit no significant negative effect on cell viability up to the concentration of 10 μM. Accordingly, we chose a series of concentrations (0.1, 1 and 10 μM) of RSV to investigate the effect on autophagy of endothelial cells. We hope to find whether RSV can affect autophagy in endothelial cells at the concentrations that cells showed no changes in cell viability. Moreover, the concentrations applied in our study were in line with some previously published works [8, 36]. Autophagy is a cellular housekeeping process involved in the turnover of cytoplasmic organelles and proteins through a lysosome-dependent degradation process, and it plays a critical role in cancers, neurodegenerative disorders, and cardiovascular diseases [37–39]. Autophagy has been described as a major cytoprotective mechanism associated with phytochemicals [40]. Additionally, RSV may attenuate endothelial inflammation by inducing autophagy [20]. Moreover, the neuroprotective effects of RSV against Parkinson’s disease and Alzheimer’s disease are due to autophagy [41, 42]. These findings strongly indicate that autophagy is critical for the multiple RSV-induced bio-protective effects. Thus, we examined the role of autophagy in RSV-induced endothelial protection in PA-stimulated HUVECs. In this study, the RSV pretreatment significantly ameliorated endothelial oxidative injury in PA-treated HUVECs, as indicated by increasing cell proliferation and decreasing ROS and MDA levels (Fig. 2). Furthermore, PA-induced oxidative injury increased the production of LC3 II and P62, implying that autophagic flux was blocked. The RSV pretreatment considerably increased and decreased the LC3 II and P62 levels, respectively, which demonstrated that RSV induces autophagic flux. However, a 3-MA treatment attenuated the RSV-induced formation of autophagosomes (Fig. 3). These results suggested that an autophagy-dependent mechanism is important for the RSV-mediated attenuation of oxidative injury in PA-stimulated HUVECs. However, the exact mechanisms underlying RSV-induced autophagy remain uncharacterized.

Settembre et al. recently reported that TFEB is a key regulator of an autophagy–lysosome signaling pathway in a starvation model. TFEB is also required to induce the expression of starvation-response genes involved in several lipid catabolism steps occurring in different cellular compartments, such as the transport of fatty acid chains and the oxidation of free fatty acids in mitochondria and peroxisomes. Previous study revealed that the TFEB overexpression may affect lipid catabolismm, inhibit etabolic activities, and subsequently ameliorate the obesity-related metabolic syndrome [16]. Thus, activating TFEB may extend the lifespan of *C. elegans*, indicating this transcription factor influences longevity [12]. We previously observed that RSV can promote longevity [43]. Therefore, we presumed that there is a relationship between RSV and TFEB. There are reports that TFEB has a global transcriptional regulatory function during lipid catabolism because it directly regulates the proliferator-activated receptor gamma coactivator-1α (PGC1α). Moreover, RSV may prevent renal lipotoxicity through the AMPK–SIRT1–PGC1α axis [44]. Furthermore, TFEB activity is regulated transcriptionally and post-transcriptionally by nutrients [16]. Therefore, we hypothesized that TFEB may be regulated by RSV. In this study, we aimed to verify whether TFEB is necessary for the RSV-induced autophagy that protects PA-treated HUVECs against oxidative injury. Previous studies have shown the activation of TFEB could be reflected by the total protein expression of the total nuclear TFEB and the ratio of nuclear TFEB/ cytosolic TFEB by western blot comprehensively [45–48]. In our study, we found 1 μM of RSV could increase the ratio of nuclear TFEB/ cytosolic TFEB but not elevate the total nuclear TFEB. However, 10 μM of RSV could significantly increase the ratio of nuclear TFEB/ cytosolic TFEB but not elevate the total nuclear TFEB. Thus, we argue that the 10 μM of RSV could trigger the nuclear translocation of TFEB, namely activating the TFEB in the endothelial cells. We observed that *TFEB* expression in HUVECs may be upregulated by RSV in a concentration-dependent manner, with 10 μM of RSV resulting in the most efficient *TFEB* expression. We also examined the expression of the downstream targets of TFEB, including *MAP 1LC3B* and *UVRAG*, which encode proteins affecting autophagy progression, and *ATP6V0D1*, *LAMP1* and *CTSB*, which encode lysosomal transmembrane proteins and lysosomal hydrolase. In addition to regulating TFEB production, RSV also induced a concentration-dependent increase in the expression of these genes in HUVECs (Fig. 4). These results indicate that *TFE*B expression is positively regulated by RSV, implying that TFEB represents a novel RSV target related to the protection against endothelial oxidative damage. As expected, the RSV-induced autophagy process and endothelial protective effect were notably attenuated when PA-treated HUVECs were transfected with *TFEB* siRNA (Figs. 5 and 6). This confirms that TFEB is indispensable for the RSV-induced autophagy in the endothelial oxidative injury model.

There are still some limitations in this study, which require further explanation. First, our data were based on isolated HUVECs in vitro, but not the more complex microenvironment in vivo. Whether RSV stimulates autophagy via activating TFEB in vivo need to be validated further. Besides, it also needs to be explored whether there is the other mechanism of anti-AS effects of RSV and which one is dominant in a different situation. This finding provides valuable clues regarding the potential protective role of RSV. We provided critical experimental evidence of the good prospect of RSV in the clinical treatment of cardiovascular diseases. It opened a view in the search of new mitochondria-targeting drugs for anti-AS therapy.

## Conclusion

**Fig. 7:**
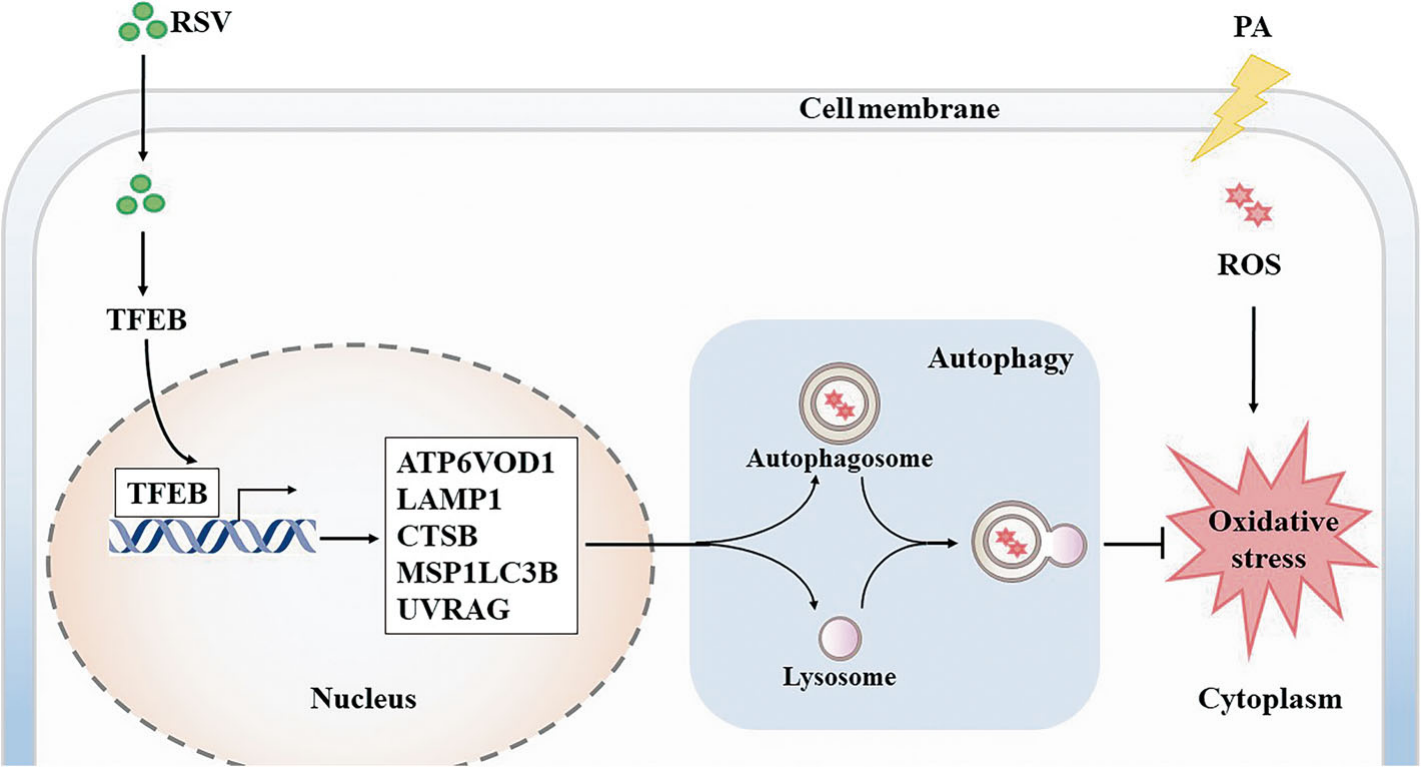
Schema of RSV-induced autophagy via activating TFEB in PA-stimulated HUVECs. Depending on the proposed signaling pathways: RSV activates TFEB and TFEB-target genes. Further, inducing the entire autophagy progress, concluding autophagosome as well as lysosome formation, ultimately, RSV attenuates PA-caused endothelial oxidative injury

To the best of our knowledge, the data presented here reveals for the first time that RSV protects HUVECs against oxidative injury caused by PA via autophagy mediated by a TFEB-dependent mechanism (Fig. 7). Our results provide new insights into the possibility that TFEB is targeted by RSV, at least partially, and suggest that the associated induced autophagy may have implications for improving cardiovascular health. These results provide critical experimental evidence of the potential utility of the clinical application of RSV for preventing and treating AS.

## Data Availability

Access to the data of this study will be considered by the corresponding author upon reasonable request.

## Abbreviations

3-MA: 3-methyladenine
AS: Atherosclerosis
ATP6V0D1: ATPase H+ Transporting V0 Subunit D1
CTSB: Cathepsin B
HUVECs: Human umbilical vein endothelial cells
LAMP-1: Lysosomal-associated membrane protein 1
MAP1LC3B: Microtubule-associated proteins 1A/1B light chain 3B
PA: Palmitic acid;ROS, reactive oxygen species
siRNA: Small interfering RNA
TFEB: Transcription factor EB
UVRAG: UV resistance-associated gene
